# The non-structural protein of SFTSV activates NLRP1 and CARD8 inflammasome through disrupting the DPP9-mediated ternary complex

**DOI:** 10.1371/journal.ppat.1013258

**Published:** 2025-07-03

**Authors:** Pan-Pan Liu, Shu-Peng Jiang, Bang Li, Wen-Tao Gui, Xiang-Rong Qin, Xue-Jie Yu

**Affiliations:** 1 State Key Laboratory of Virology, School of Public Health, Wuhan University, Wuhan, China; 2 Department of Clinical Laboratory, Renmin Hospital of Wuhan University, Wuhan, China; 3 Department of Genetics and Microbiology, Faculty of Science, Charles University, Prague, Czech Republic; 4 Department of Clinical Laboratory, The Second Hospital of Shandong University, Jinan, China; University of Washington School of Medicine, UNITED STATES OF AMERICA

## Abstract

Inflammasomes function as immune-signaling platforms that were assembled following detection of pathogens. NLRP1 and CARD8 are related inflammasomes that use their C-terminal (CT) fragments containing a caspase recruitment domain (CARD) and the UPA domain to initiate the inflammasome. At rest, dipeptidyl peptidases 8 and 9 (DPP8/9) inhibit inflammatory CT by interacting with the function-to-find domain (FIIND) of NLRP1/CARD8 and forming an inhibitory NLRP1/CARD8-DPP9 ternary complex consisting of DPP9, full-length NLRP1/CARD8, and NLRP1/CARD8 CT. However, the specific triggers of NLRP1 and CARD8 have not yet been fully identified. Here, we report that a tick-borne bunyavirus SFTSV infection activates the NLRP1 inflammasome in primary keratinocytes and the CARD8 inflammasome in macrophages in a similar manner by targeting the ternary inhibitory complex, respectively. Mechanistically, SFTSV NSs interact with NLRP1 and CARD8 via their FIIND domains, suggesting that DPP8/9 are likely to compete for binding; on the other hand, NSs promote the degradation of DPP8 and DPP9. Both contribute to more efficient destabilization of the DPP8/9 ternary complex and release the activated CT. Moreover, CARD8 deletion promotes SFTSV replication. In conclusion, we found a novel mechanism of viral protein activation of NLRP1 and CARD8 by disrupting the DPP9-binding checkpoint.

## Author summary

Severe fever with thrombocytopenia syndrome (SFTS) is a viral hemorrhagic fever caused by a tick-borne bunyavirus SFTSV with a mortality rate of up to 30%. Inflammasomes are multiprotein complexes formed in response to pathogens. NLRP1 and CARD8 are related germline-encoded pattern recognition receptors that form inflammasomes, but their activation mechanisms have not yet been fully established. At rest, NLRP1 and CARD8 are kept in an inactive state by binding to DPP8/9 (dipeptidyl peptidase 8 and 9). Here, we discover a novel cognate activation mechanism for NLRP1 and CARD8 by disrupting the DPP9-binding checkpoint. In our study, we show that SFTSV infection activates the NLRP1 inflammasome and the CARD8 inflammasome in a similar manner by targeting the ternary inhibitory complex. Moreover, we show that CARD8 are important in the control of SFTSV infection.

## Introduction

The host innate immune system uses germline-encoded pattern-recognition receptors to detect pathogen-associated molecular patterns (PAMPs) and danger-associated molecular patterns (DAMPs) [1]. Nucleotide-binding domain leucine-rich repeat proteins (NLRs) belong to a large family of pattern recognition receptors that nucleate and assemble into inflammasome complexes upon activation [2]. The canonical inflammasome complex minimally consists of an NLR sensor, the adaptor protein ASC (apoptosis-associated speck-like protein containing a CARD, also known as PYCARD), and the effector inflammatory caspase-1 [3]. Assembly of an inflammasome leads to caspase-1 activation, the secretion of proinflammatory cytokines such as interleukin-1β (IL-1β) and IL-18, and gasdermin D (GSDMD)-dependent pyroptotic cell death [3].

At least 22 different kinds of NLRs are present in humans, which vary in terms of tissue distribution. Similar to other NLRPs, NLRP1 harbors three characteristic domains: an N-terminal death-fold domain, a central nucleotide-binding or NACHT domain, and a C-terminal leucine-rich repeat domain (LRR) [1,3]. A unique feature of NLRP1 is that it has two additional domains at its C terminus, a FIIND (function-to-find domain) and a CARD [1,2,4]. CARD8 has an N-terminal disordered stretch of ∼160 amino acids followed by a similar FIIND-CARD region. The NLRP1 and CARD8 FIINDs undergo autoproteolysis between their ZU5 (ZO-1 and UNC5) and UPA (conserved in UNC5, PIDD, and ankyrin) subdomains, generating N-terminal (NT) and C-terminal (CT) fragments that remain non-covalently associated in an autoinhibited state [2–5]. In addition, the CARD domain of NLRP1 or CARD8 triggers inflammasome assembly differently from the PYD domain of other PRRs [3]. CARD8 cannot induce ASC oligomerization and, therefore, functions in an ASC-independent manner. Instead, CARD8 directly engages CASP1 upon activation, while human NLRP1 requires ASC to bridge interactions with CASP1. NLRP1 is highly polymorphic both within and across species. Humans have only one NLRP1 protein, but rodents possess several paralogues, including NLRP1A, NLRP1B, and NLRP1C in mice, which lack the N-terminal PYD domain [1,4,6]. However, neither rodent species is homologous to CARD8.

In resting cells, NLRP1 and CARD8 are kept in an inactive state by dipeptidyl peptidase 8 and 9 (DPP8 and DPP9). The DPP9 acts as a negative regulator of the NLRP1/CARD8 and restrains its activation by binding to the NLRP1/CARD8 FIIND domain [7–9]. Furthermore, the cryo-EM structure studies revealed that DPP9 forms a ternary complex (NLRP1 ^FL^/CARD8^FL^-DPP9-NLRP1^CT^/CARD8^CT^) with full-length NLRP1/CARD8 and the NLRP1/CARD8 CT to sequester the bioactive CT, preventing inflammasome activation [8–10]. The DPP8/DPP9 inhibitor VbP activates the NLRP1 and CARD8 inflammasome by weakening the NLRP1-DPP9 interaction and accelerating the degradation of the N-terminal fragment [7,8,10]. Several unrelated danger-associated signals induce the proteasome-mediated degradation of the NT fragments of CARD8 and/or NLRP1, thereby releasing free CT fragments that can assemble into inflammasomes. Recently, enteroviral 3C proteases, long dsRNA, cytosolic peptide accumulation, and reductive stress were identified as the natural triggers for the human NLRP1 inflammasome [3,11,12]. This occurs through a process of “functional degradation” in a proteasome-dependent manner [13]. In a similar fashion, CARD8 is a broad sensor of viral protease activities [14–16].

Severe fever with thrombocytopenia syndrome (SFTS) is an emerging tick-borne hemorrhagic fever caused by SFTSV, also known as *Dabie bandavirus*, first reported in Eastern China in 2009 and subsequently found in South Korea and Japan [17–19]. The major clinical symptoms of SFTS are fever, malaise, myalgia, arthralgia, thrombocytopenia, and leukopenia [20–23]. In severe cases, patients may develop severe hemorrhagic fever with symptoms including intracerebral hemorrhage, gastrointestinal bleeding, and multiple organ failure [20–22]. In addition, SFTSV is associated with mortality rates as high as 30%, and there are no specific antiviral drugs or effective vaccines [19,24]. The World Health Organization declared SFTSV as a priority pathogen in 2018 [21,22]. In SFTS patients, a “cytokine storm” is considered the main pathophysiological feature of severe and fatal disease along with hemorrhagic complications arising from thrombocytopenia [22]. Lethal SFTS is associated with high levels of proinflammatory cytokines, including IL-1β, IL-6, and IFNγ [21,22,25]. Viral immunopathology can be targeted in two ways: either by inhibiting virus replication or by dampening the inflammatory response [22,26]. Despite this, how SFTSV infection leads to inflammasome activation is not clearly understood.

The SFTSV genome contains three single-stranded negative-sense RNAs: the large (L), medium (M), and small (S) segments. The L segment encodes the RNA-dependent RNA polymerase (RdRp), the M segment encodes the viral glycoprotein precursor, which is processed into Gn and Gc, and the S segment encodes the nucleocapsid protein (Np) and a nonstructural protein (NSs) [27]. NSs play an important role in antiviral IFN response for immune evasion [28].

In this study, we found that SFTSV infection activates the NLRP1 inflammasome in primary keratinocytes and the CARD8 inflammasome in macrophages in a similar manner by targeting the ternary inhibitory complex, respectively. SFTSV activates the NLRP1 inflammasome and CARD8 inflammasome via two distinct mechanisms. First, SFTSV infection promotes NLRP1/CARD8 N-terminal functional degradation through an unknown mechanism. Second, SFTSV infection disrupts the stability of the NLRP1/CARD8-DPP8/9 inhibitory ternary complex. Mechanistically, on the one hand, SFTSV NSs interacts with NLRP1/CARD8 FIIND and form a competitive binding with DPP9; on the other hand, NSs promotes the degradation of DPP8 and DPP9. We further show that CARD8 is the principal inflammasome sensor for SFTSV infection-induced pyroptosis. Genetic removal of CARD8 alleviates SFTSV-induced cell death and cytokine release, significantly increasing viral replication. Our work elucidates that SFTSV is a trigger of the NLRP1 and CARD8 inflammasome and highlights the potential therapeutic benefit of activating CARD8 in macrophages.

## Results

### SFTSV infection activates the NLRP1 inflammasome in primary keratinocytes

**Fig 1:**
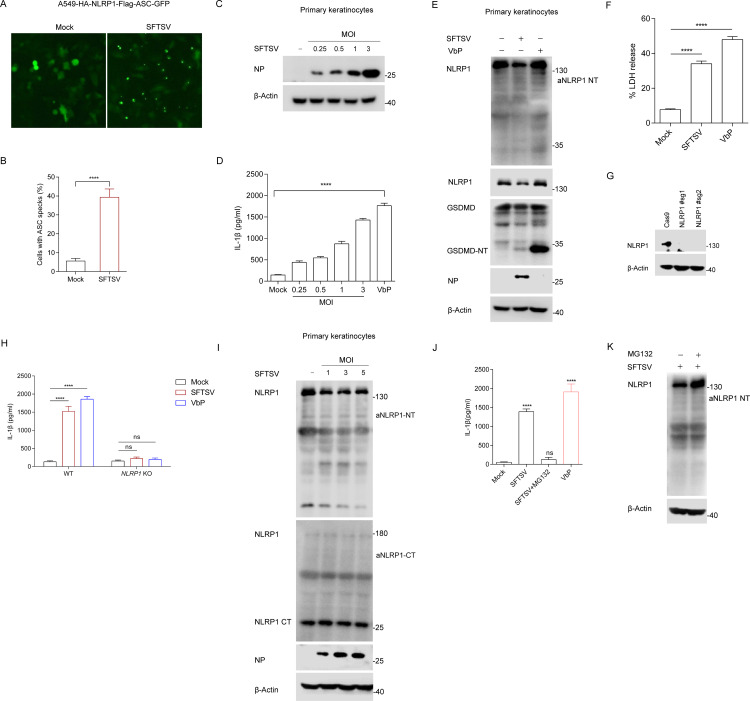
SFTSV induces NLRP1 inflammasome activation in primary keratinocytes. (A-B) ASC specks fluorescence microscopy images (**A**) and quantification (**B**) of A549-HA-NLRP1-Flag-ASC-GFP cells infected with SFTSV or without SFTSV (MOI = 0.5) for 24 h. Scale bar, 100 μm. (C-D) Detection of SFTSV NP (**C**) and IL-β production (**D**) in primary keratinocytes infected with SFTSV at different MOIs or stimulated with VbP (2 μM) for 24 h. (E-F) GSDMD cleavage (**E**) and LDH release (**F**) in primary keratinocytes infected with SFTSV (MOI = 1) or stimulated with VbP (5 μM) for 36 h. (G) Expression of NLRP1 in primary keratinocytes treated with lentivirus-mediated CRISPR-Cas9 and *NLRP1*-specific single-guide RNA (sgRNA) or the non-targeting sgRNA control. (H) IL-1β production in wild-type or NLRP1-deficient primary keratinocytes infected with SFTSV (MOI = 1) or stimulated with VbP (2 μM) for 24 h. (I) Primary keratinocytes were infected with SFTSV at different MOIs for 24 h, endogenous NLRP1 was detected using N-terminal and C-terminal antibodies with Western blot, respectively. (J-K) Primary keratinocytes were infected with SFTSV (MOI = 1) and treated with MG132 (2 μM) for 24 h, IL-1β release (**J**) in the cell supernatant was measured with ELISA; endogenous NLRP1 (**K**) was detected with Western blot. All data represent three independent experiments and presented as mean±s.d. *P < 0.05, **P < 0.01, ***P < 0.001, ****P < 0.0001, *ns*, not significant. For statistical analysis, one-way ANOVA in (D, J), two-tailed unpaired Student’s *t*-test in (B, F, H).

To test whether SFTSV can activate the NLRP1 inflammasome, we first constructed an A549 cell line stably expressing HA-NLRP1-Flag and ASC-GFP (A549-HA-NLRP1-Flag-ASC-GFP cells). The recombinant NLRP1 inflammasome system was established using HEK293T cells, in which cells were co-transfected with plasmids encoding NLRP1, ASC, pro-CASP1, and pro-IL-1β (ASC-caspase-1-pro-IL-1β HEK293T). Upon activation, the assembly of NLRP1 inflammasome led to ASC oligomerization, ASC speck formation, and processing of IL-1β into the p17 mature forms, which were the direct indicators of inflammasome activation. In the recombinant NLRP1 inflammasome system, pro-IL-1β was cleaved into activated IL-1β after SFTSV infection (S1A Fig). Infection with SFTSV also triggered ASC oligomerization and ASC speck formation (Figs 1A,1B, and S1B). These data suggest that SFTSV activates the reconstituted NLRP1 inflammasome.

It is increasingly recognized that different tissues and cell types employ distinct repertoires of NLR sensors [16]. Primary keratinocytes express the functional NLRP1 inflammasome and do not contain NLRP3, which is almost exclusively expressed by macrophages. Next, we studied whether the endogenous NLRP1 inflammasome is activated upon infection with SFTSV in primary keratinocytes. We first determined that SFTSV infected efficiently in primary keratinocytes (Fig 1C). NLRP1 activation was assessed by measuring GSDMD cleavage and IL-1β release in SFTSV-infected primary keratinocytes. SFTSV infection also led to significant IL-1β release, GSDMD cleavage, and LDH release, as observed with VbP treatment (Fig 1D–1F). To further investigate whether NLRP1 was responsible for SFTSV-mediated inflammasome activation, we utilized the CRISPR-Cas9 technique to construct NLPR1-KO (NLRP1^-/-^) primary keratinocytes (Fig 1G). We observed that SFTSV infection leads to a robust release of IL-1β, which is blunted in the absence of NLRP1 (Fig 1H).

Notably, the endogenous NLRP1 NT was reduced in primary keratinocytes following SFTSV infection (Figs 1E,1I, and S1C), suggesting a mechanism of infection-induced degradation. In A549 cells stably expressing NLRP1, SFTSV infection also reduced NLRP1 NT (S1D and S1E Fig). In light of these results, SFTSV infection may trigger NLRP1 N-terminal degradation. There is a general mechanism by which the activation of NLRP1 inflammasomes involves functional degradation [29]. In addition, SFTSV-induced IL-1β release in primary keratinocytes was sensitive to proteasome inhibitor MG132 and NEDD8 activating enzyme inhibitor MLN4924 (Figs 1J and S1F). Moreover, NLRP1 NT was restored after MG132 or MLN4924 treatment (Figs 1K,S1G, and S1H). These results suggest that NLRP1 degradation, involving cullin ubiquitin ligases and the proteasome, was necessary to activate NLRP1 after SFTSV infection, similar to other NLRP1 activators. Ultraviolet B irradiation, bacterial ribotoxins, and alphavirus infection have also been reported to induce phosphorylation in the NT-NLRP1 linker region by promoting ribotoxic stress and activating the mitogen-activated protein kinase (MAPK) ZAKα to activate the NLRP1 inflammasome [30,31]. We next tested whether NLRP1 activation by SFTSV required p38 activity. Surprisingly, phosphorylated p38 was detected in SFTSV-infected primary keratinocytes, which was significantly lower than p38 phosphorylation in ANS-treated cells (S2A and S2B Fig). To test whether SFTSV-induced NLRP1 inflammasome response relied on the p38 signaling, we tested GSDMD cleavage and IL-1β release in primary keratinocytes treated with p38α/β inhibitor SB202190 (SB), or the pan p38 inhibitor doramapimod (Dora). Our results showed that in the presence of SB or Dora, ANS and SFTSV-induced GSDMD cleavage and IL-1β production were almost completely inhibited (S2B and S2C Fig). In line with that, phosphorylation of p38 after ANS treatment was largely prevented by both p38 inhibitors (S2B Fig). However, both SB and Dora strongly decreased SFTSV infection (S2B Fig). Therefore, we could not access whether p38 kinases was actually triggered in SFTSV-mediated NLRP1 response. Overall, these findings indicated that SFTSV can activate the NLRP1 inflammasome in primary keratinocytes.

### SFTSV infection activates the CARD8 inflammasome in macrophages

**Fig 2:**
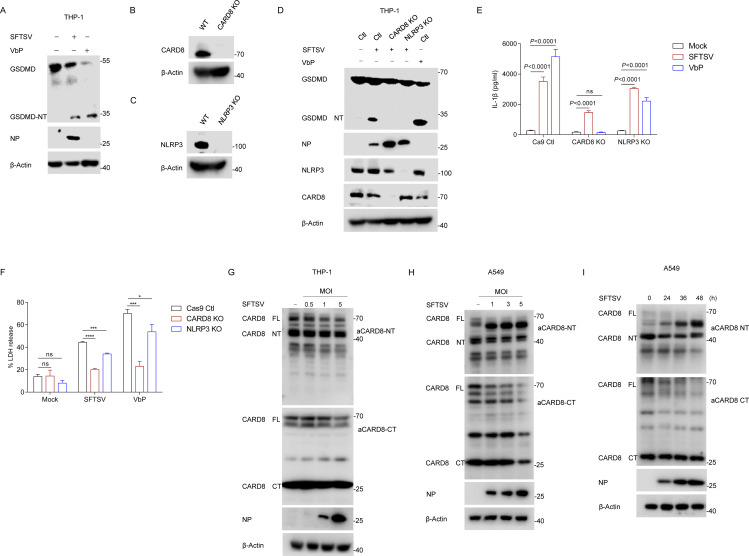
SFTSV activates the CARD8 inflammasome in macrophages. (A) GSDMD cleavage in THP-1 cells infected with SFTSV (MOI = 1) or stimulated with VbP (2 μM) for 24 h. (B) Knockout of CARD8 in THP-1 cells identified with Western blot. (C) Knockout of NLRP3 in THP-1 cells identified with Western blot. (D-F) GSDMD cleavage (D), IL-1β production (**E**) and LDH release (**F**) in THP-1 WT, CARD8 KO and NLRP3 KO infected with SFTSV (MOI = 1) or stimulated with VbP (5 μM) for 24 h. (G) THP-1 cells were infected with SFTSV at different MOIs for 24 h, endogenous CARD8 was detected using N-terminal and C-terminal antibodies with Western blot, respectively. (H) A549 cells were infected with SFTSV at different MOIs for 24 h, endogenous CARD8 was detected using N-terminal and C-terminal antibodies with Western blot, respectively. (I) A549 cells were infected with SFTSV (MOI = 1) at indicated time, endogenous CARD8 was detected using N-terminal and C-terminal antibodies with Western blot, respectively. All data represent three independent experiments and presented as mean±s.d. *P < 0.05, **P < 0.01, ***P < 0.001, ****P < 0.0001, *ns*, not significant. For statistical analysis, two-tailed unpaired Student’s *t*-test in (E, F).

CARD8 and NLRP1, which are related in their FIIND-mediated autoprocessing, DPP9-mediated suppression, and DPP8/9 inhibitor-mediated activation, share important similarities [9]. According to a previous study, transcriptomic analysis of blood samples from SFTS patients showed a strong correlation between inflammatory response and disease progression [32]. CARD8 is highly expressed in hematopoietic cells, including T cells, B cells, monocytes and macrophages [33]. We next asked whether SFTSV activates the CARD8 inflammasome in macrophages. Previous studies have found that SFTSV activated NLRP3 inflammasome and induces an inflammasome response [32,34]. To this end, we utilized CRISPR-Cas9 to delete CARD8 and NLRP3 in THP-1 cells, respectively. Cleaved GSDMD was detected in THP-1 cells following SFTSV infection (Fig 2A), demonstrating inflammasome activation. Both CARD8 KO and NLRP3 KO abrogated GSDMD cleavage (Fig 2B–2D). Consistent with previous studies, NLRP3 KO indeed reduced SFTSV-induced IL-1β secretion (Fig 2E). However, CARD8 KO reduced IL-1β secretion to a greater extent than the NLRP3 KO (Fig 2E). This effect was particularly evident for LDH release, which was largely eliminated in CARD8 KO (Fig 2F), confirming previous findings that CARD8 activation is more pro-death. Together, CARD8 is also activated by SFTSV in THP-1 cells and might play even more important role in SFTSV-mediated inflammasome activation compared to NLRP3. Both NLRP1 and CARD8 use a FL FIIND and DPP9 to capture the active CT. Similarly, in both SFTSV-infected THP-1 and A549 cells, we observed that endogenous CARD8 FL/NT was reduced in either an MOI dose-dependent manner or time-dependent manner (Fig 2G–2I). SFTSV infection, therefore, increases the ratio of C-terminal/N-terminal fragments, which is predicted to disrupt ternary complex formation leading to CARD8 inflammasome activation. Overall, these findings indicated that SFTSV can activate the CARD8 inflammasome in macrophages.

### SFTSV NSs mediates the activation of NLRP1 and CARD8 inflammasome

**Fig 3:**
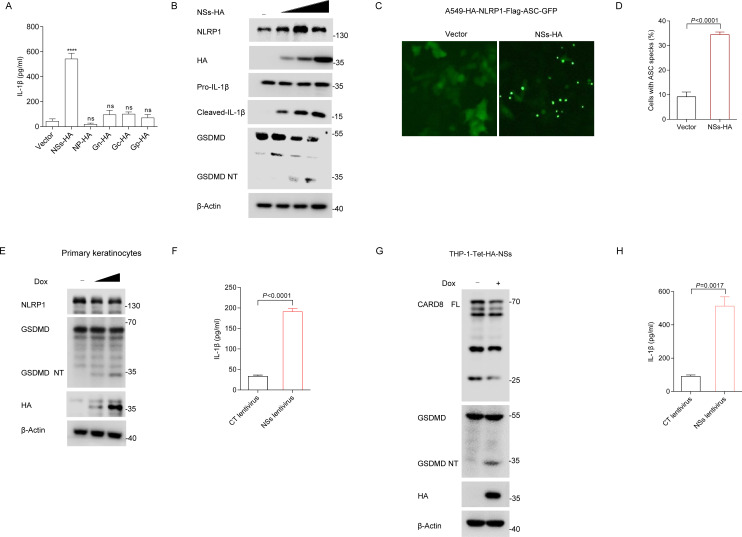
The role of SFTSV NSs in the activation of the NLRP1 and CARD8 inflammasome. (A) ASC-caspase-1-pro-IL-1β HEK293T cells were transfected with indicated expression vector for 36 h. Supernatants were analyzed for IL-1β with ELISA. (B) ASC-caspase-1-pro-IL-1β HEK293T cells were transfected with plasmids NSs, production of p17 and GSDMD processing were detected with Western blot. (C-D) ASC specks fluorescence microscopy images (**C**) and quantification (**D**) of A549-HA-NLRP1-Flag-ASC-GFP cells transfected NSs-HA for 24 h. Scale bar, 100 μm. (E-F) GSDMD cleavage (**E**) and IL-1β (**F**) production in primary keratinocytes expressing Tet-HA-NSs and treated with doxycycline (1 μg/ml) for 48 h. (G-H) GSDMD cleavage (**G**) and IL-1β (**H**) production in THP-1 cells expressing Tet-HA-NSs and treated with doxycycline (1 μg/ml) for 48 h. All data represent three independent experiments and presented as mean±s.d. *P < 0.05, **P < 0.01, ***P < 0.001, ****P < 0.0001, *ns*, not significant. For statistical analysis, two-tailed unpaired Student’s *t*-test in (A, D, F, H).

Negative-strand RNA viruses that contain blunt short double-strand 5’ triphosphate RNA in the panhandle region of their single-stranded genome, but lack long double-strand RNA [35]. During infection, SFTSV produces 5’ triphosphate RNA, which primarily activates RIG-I, not MDA5 to trigger the host responses [36]. Previous studies have found that only long dsRNA (>500 bp) can activate the NLRP1 inflammasome [3]. To determine how SFTSV activates the NLRP1 inflammasome, we expressed all 5 SFTSV proteins using an exogenous recombinant NLRP1 inflammasome system to detect the release of IL-1β. ELISA results showed that NSs caused the highest amount of IL-1β release (Fig 3A), indicating that NSs is the major factor of SFTSV to induce IL-1β secretion. Next, we further analyzed whether NSs played a role in inflammasome activation. In the recombined NLRP1 inflammasome system, expression of NSs caused the production of mature IL-1β (p17), cleavage of GSDMD in dose-dependent manners (Fig 3B), suggesting that NSs plays an important role in the activation of NLRP1 inflammasome. In A549-HA-NLRP1-Flag-ASC-GFP cells, the expression of NSs triggered ASC speck formation (Fig 3C and 3D).

In addition, we also examined whether NSs are capable of activating the endogenous NLRP1 and CARD8 inflammasome. Then, we generated the primary keratinocytes cell lines stably expressing NSs under a doxycycline-inducible promoter. We found that expression of NSs in primary keratinocytes promotes the reduction of endogenous NLRP1 NT and leads to the cleavage of GSDMD and IL-1β release (Fig 3E and 3F). Similarly, using a doxycycline-inducible expression system, we showed that expression of NSs in THP-1 also promotes the reduction of endogenous CARD8 FL and leads to the cleavage of GSDMD, IL-1β release (Fig 3G and 3H). Collectively, these findings indicated that SFTSV NSs facilitates the activation of NLRP1 and CARD8 inflammasome.

### SFTSV NSs interacts with the FIIND domains of NLRP1 and CARD8

**Fig 4:**
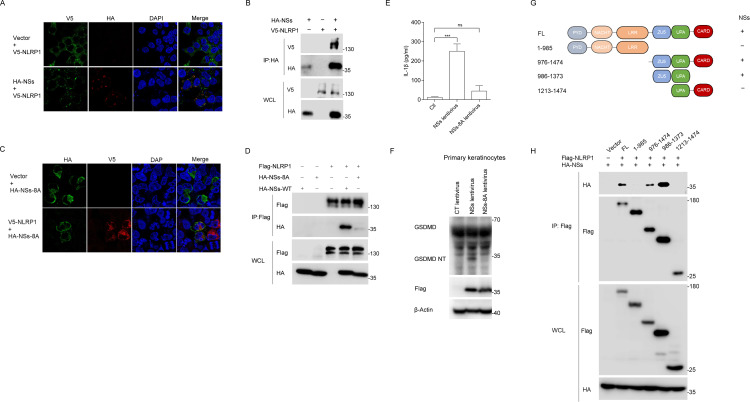
SFTSV NSs interacts with NLRP1. (A) Colocalization of NLRP1 and NSs in HEK293T cells co-transfected with V5-NLRP1 and HA-NSs for 48 h. Scale bar is 20 μm.(**B**) Co-IP assay between V5-NLRP1 and HA-NSs in HEK293T cells transfected with the indicated expression vectors for 48 h. (C) Colocation of NLRP1 and NSs-8A in HEK293T cells co-transfected with V5-NLRP1 and HA-NSs-8A for 48 h. Scale bar is 20 μm. (D) Co-IP assay between HA-NSs or HA-NSs-8A and Flag-NLRP1 in HEK293T cells transfected with the indicated expression vectors for 48 h. (E-F) IL-1β production (**E**) and GSDMD cleavage (**F**) in primary keratinocytes infected with lentiviruses expression Flag-NSs-WT or Flag-NSs-8A or carrying vector for 48 h. (G) Summary of mapping experiments to identify the NSs-binding domain in NLRP1. (H) Co-IP assay between NSs and NLRP1 domain truncation in HEK293T cells transfected with the indicated expression vectors for 48 h. All data represent three independent experiments and presented as mean±s.d. *P < 0.05, **P < 0.01, ***P < 0.001, ****P < 0.0001, *ns*, not significant. For statistical analysis, two-tailed unpaired Student’s *t*-test in (E).

To characterize the molecular mechanism of NSs activating the NLRP1 inflammasome, we investigated whether NSs can interact with NLRP1. In HEK293T cells, overexpressed NLRP1 was mainly diffusely distributed in the cytoplasm, but when co-expressing with NSs, NLRP1 and NSs were found to be colocalized together in the cytoplasm (Fig 4A). The structure where NSs and NLRP1 co-localized appears to be viroplasm-like structures formed by NSs [37,38]. Moreover, when NSs were overexpressed in Hela cells, confocal results indicated that NSs were co-localized with endogenous NLRP1 as well (S3A Fig). Furthermore, co-immunoprecipitation (Co-IP) assays showed that NSs, but not NP, interacted with NLRP1 (Figs 4B,S3B, and S3C). These results demonstrate that NSs interact specifically with NLRP1.

Next, we constructed several mutants of NSs to determine which region of NSs was required for NLRP1 binding and inflammasome activation. There is evidence that the NSs can form inclusion bodies that isolate some immune proteins of the host to evade immune response [36,39]. This has been shown to be an important virulence factor of SFTSV. It was found that NSs-8A mutants carrying alanine substitutions for the eight amino acids S97xLRWPxG104 could not form inclusion bodies [40] (Fig 4C). Compared to wild-type NSs (NSs-WT), the interaction between NSs-8A and NLRP1 was significantly weakened (Fig 4C and 4D). In addition, NSs-8A hardly induce mature IL-1β production in the recombinant NLRP1 inflammasome systems (S3D Fig). Consistently, unlike NSs-WT, overexpression of NSs-8A by lentivirus did not induce mature IL-1β production and GSDMD cleavage in primary keratinocytes (Fig 4E and 4F). These findings suggested the direct correlation between the inclusion body formation and the NLRP1 inflammasome-activation ability of NSs. In addition, we constructed a series of NLRP1 truncations to understand the interaction domains between NLRP1 and NSs (Fig 4G). Co-IP assays showed that the FIIND region interacts predominantly with NSs (Fig 4H).

**Fig 5:**
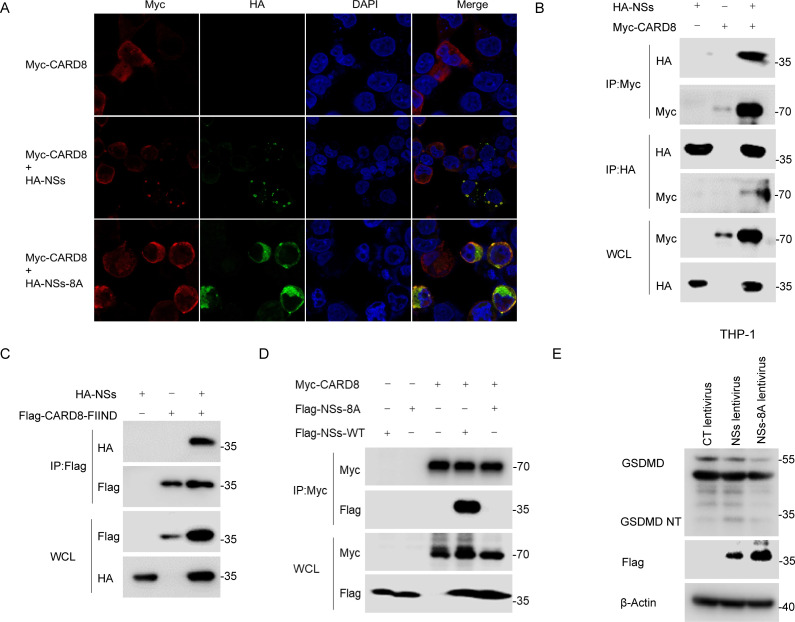
SFTSV NSs interacts with CARD8. (A) Colocalization of NSs, NSs-8A and NLRP1 in HEK293T cells co-transfected with HA-NSs, HA-NSs-8A and V5-NLRP1 for 48 h. Scale bar is 10 μm. (B) Co-IP assay between HA-NSs and Myc-CARD8 in HEK293T cells transfected with the indicated expression vectors for 48 h. (C) Co-IP assay between HA-NSs and Flag-CARD8-FIIND in HEK293T cells transfected with the indicated expression vectors for 48 h. (D) Co-IP assay between Flag-NSs or Flag-NSs-8A and Myc-CARD8 in HEK293T cells transfected with the indicated expression vectors for 48 h. (E) GSDMD cleavage in THP-1 cells infected with lentiviruses expression Flag-NSs-WT or Flag-NSs-8A or carrying vector for 48 h.

CARD8 and NLRP1 are related inflammasomes with unique FIIND domains. We speculate that CARD8 may also interact with NSs through the FIIND domain. In HEK293T cells, confocal results showed that NSs were co-localized with CARD8 (Fig 5A). The Co-IP assays further determined the interaction between NSs and CARD8 by FIIND domain (Fig 5B and 5C). Similarly, NSs-8A mutant had a significantly attenuated interaction with CARD8 compared to NSs-WT (Fig 5A and 5D). In addition, GSDMD cleavage induced by NSs-8A was significantly reduced compared to NSs-WT in THP-1 cells (Fig 5E). Collectively, these results suggest that NSs interacts with NLRP1 and CARD8 respectively by their FIIND domain.

### NSs impairs the interaction of NLRP1/CARD8 and DPP9

**Fig 6:**
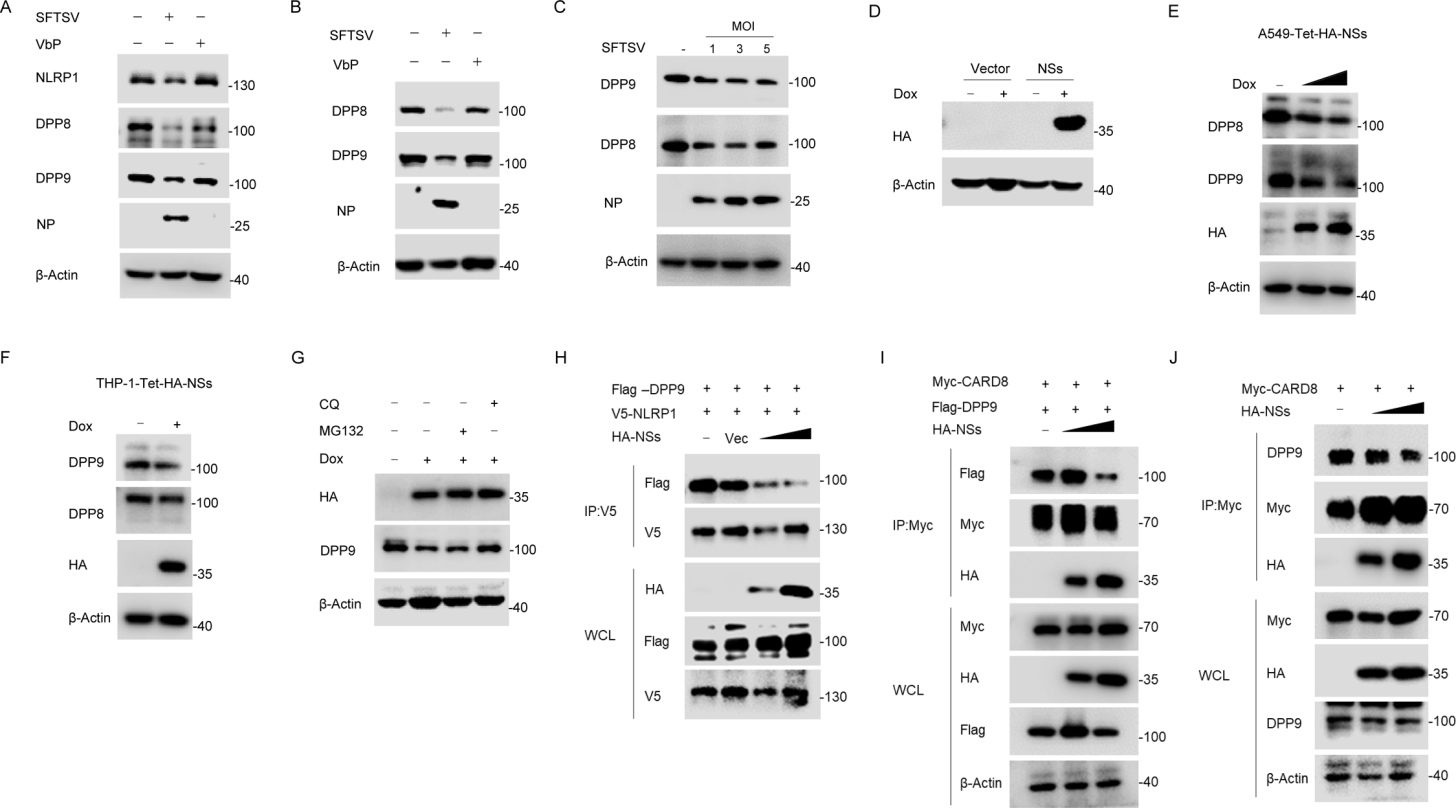
NSs impairs the interaction of NLRP1/CARD8 and DPP9. (A) Primary keratinocytes were infected with SFTSV (MOI = 1) or stimulated with VbP (5 μM) for 36 h, endogenous DPP8 and DPP9 were detected with Western blot. (B) THP-1 cells were infected with SFTSV (MOI = 1) or stimulated with VbP (2 μM) for 24 h, endogenous DPP8 and DPP9 were detected with Western blot. (C) A549 cells were infected with different MOI SFTSV for 24 h, endogenous DPP8 and DPP9 were detected with Western blot. (D) A549 cells expressing Tet-HA-NSs were treated with doxycycline (1 μg/ml) for 48 h, and identified with Western blot. (E) A549-Tet-HA-NSs cells were treated with doxycycline (1 μg/ml) for 48 h, endogenous DPP8 and DPP9 were detected with Western blot. (F) THP-1-Tet-HA-NSs cells were treatment with doxycycline (1 μg/ml) for 48 h, endogenous DPP8 and DPP9 were detected with Western blot. (G) A549-Tet-HA-NSs cells were treatment with doxycycline (1 μg/ml) for 48 h and then treated with MG132 (10 μM) or CQ (50 μM) for 6 h before harvest. (H) Co-IP of NLRP1-DPP9 with NSs in HEK293T cells transfected with indicated expression vectors for 48 h. (I) Co-IP of CARD8-DPP9 with NSs in HEK293T cells transfected with indicated expression vectors for 48 h. (J) Co-IP of CARD8-DPP9 with NSs in HEK293T cells transfected with Myc-CARD8 and HA-NSs for 48 h.

CARD8 and NLRP1 are related inflammasomes repressed by the enzymatic activities and protein structures of the DPP8/9. The structures reveal a ternary complex that comprises DPP9, FL NLRP1/CARD8 and the NLRPT/CARD8 CT. DPP8/9 appears to be somehow connected to the primordial function of NLRP1 inflammasome [41,42]. Of note, we observed the reduced abundance of both DPP8 and DPP9 in SFTSV-infected primary keratinocytes (Fig 6A). To further investigate this phenomenon, we tested it on multiple cell types. As expected, SFTSV infection led to the loss of both DPP8 and DPP9 in THP-1 and A549 cells (Fig 6B and 6C). Next, we used lentivirus expressing human DPP9 in primary keratinocytes to evaluate whether overexpression of DPP9 could modulate NLRP1 response to SFTSV. Our results showed that overexpression of DPP9 significantly attenuates GSDMD cleavage and IL-1β release induced by SFTSV infection in primary keratinocytes (S4A and S4B Fig). Consistently, overexpression of DPP9 almost completely inhibited the cleavage of pro-IL-1β by SFTSV infection in HEK293T recombinant NLRP1/CARD8 system (S4C and S4D Fig). These results suggest that SFTSV infection may target the destruction of inhibitory ternary complex formation, leading to NLRP1 or CARD8 inflammasome activation.

We further characterized the virus protein molecules that involved in the loss of DPP8 and DPP9. Using a doxycycline-inducible expression system, we showed that NSs decreased the abundance of DPP9 and DPP8 in both A549 and THP-1 cells (Fig 6D–6F). Moreover, autophagy inhibitors chloroquine (CQ) treatment but not MG132 rescued the degradation of DPP9 induced by NSs (Fig 6G), indicating that NSs may degrade DPP9 through autophagy pathway. Furthermore, cleavage of pro-IL-1β caused by NSs was attenuated to some extent by CQ in HEK293T recombinant NLRP1 system (S4E Fig). Our study discovered that NSs and DPP9 interact with NLRP1 and CARD8 via their FIIND domain. Next, we examined whether NSs affected the interaction of NLRP1/CARD8 with DPP9. Indeed, Co-IP assays showed that DPP9 binding was weakened by increasing NSs expression (Fig 6H–6J), although the expression of NSs affected the abundance of CARD8 to some extent. Based on these observations, NSs likely promoted the NLRP1/CARD8 inflammasomes by disrupting the DPP9-binding checkpoint in cells.

### CARD8 deletion promotes SFTSV replication

**Fig 7:**
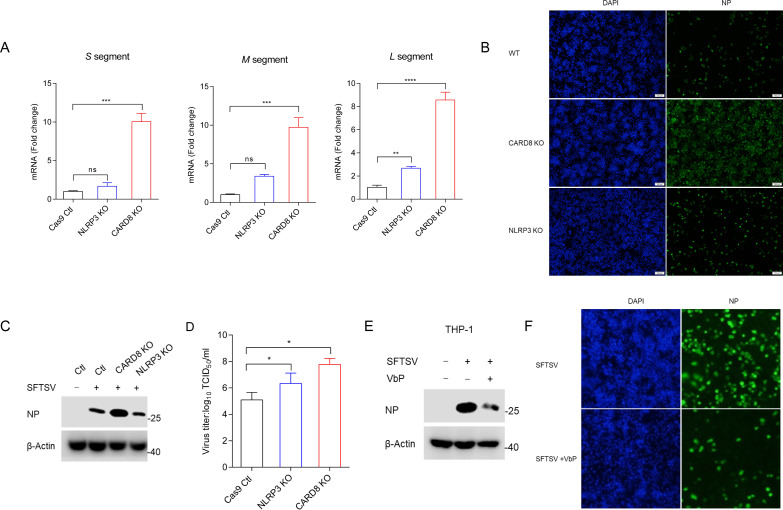
CARD8 deletion promotes SFTSV replication. (A-D) THP-1-Ctl, CARD8 KO, and NLRP3 KO were infected with SFTSV (MOI = 1) for 48 h, the mRNA levels (**A**) of SFSTV S, M, L segments were detected with RT-qPCR; (**B**) cells were stained with SFTSV NP and analyzed with immunofluorescence assays. Scale bar, 100 μm; (C) Expression of SFTSV NP was analyzed with Western blot; (D) Functional titers of SFTSV in Ctl, CARD8-KO and NLRP3-KO THP-1 cells measured by TCID_50_. (E-F) THP-1 cells were infected with SFTSV (MOI = 1) and treated with VbP (2 μM) for 24 h. (E) Expression of SFTSV NP was analyzed with Western blot; (**F**) cells were stained with SFTSV NP and analyzed with immunofluorescence assays. Scale bar, 100 μm. All data represent three independent experiments and presented as mean±s.d. *P < 0.05, **P < 0.01, ***P < 0.001, ****P < 0.0001, *ns*, not significant. For statistical analysis, two-tailed unpaired Student’s *t*-test in (A, D).

CARD8 and NLRP1 exhibit distinct expression patterns and likely have different cellular functions. Since CARD8 is expressed in SFTSV-target cells, the next question was whether the activation of CARD8 inflammasome affects viral replication. We first tested the effect of CARD8 inflammasome to SFTSV propagation by measuring the viral RNA level of SFTSV L/M/S segments. Compared to NLRP3 KO, CARD8 KO significantly increased the mRNA levels of intracellular SFTSV S, M, and L segments in THP-1 cells (Fig 7A). Correspondingly, the green fluorescence of SFTSV NP and the protein level of NP were significantly increased in CARD8 KO cells following SFTSV infection (Fig 7B and 7C). This was accompanied by increased viral propagation, as evidenced by the increased functional viral titer in the supernatant of infected cells (Fig 7D). These results showed that CARD8 KO reduced LDH release to a greater extent than the NLRP3 KO. Therefore, we hypothesized that CARD8 KO promotes viral replication primarily by inhibiting pyroptosis. Indeed, the VbP treatment significantly reduced the protein level of SFTSV NP and the green fluorescence of SFTSV NP (Fig 7E and 7F). As such, these results indicated that CARD8 deletion promotes SFTSV replication.

## Discussion

The Nod-like receptor proteins act as a critical component of innate immunity to detect a variety of pathogen-derived molecular patterns to induce an inflammatory immune response by triggering pyroptosis and cytokine release. Notably, both NLRP1 and CARD8 harbor a unique FIIND that auto-proteolyses into noncovalently associated subdomains, and proteasomal degradation of the repressive N-terminal fragment release its inflammatory C-terminal [3,6,11,13]. CARD8 and NLRP1 may have evolved to respond to different pathogens. Studies have found that human rhinovirus 3C protease and HIV protease directly cleave and activate human NLRP1 and CARD8, respectively [14,16]. Diverse pathogenic signals and the cellular consequence of DPP8/9 inhibition induce the N-terminal degradation of NLRP1 and CARD8, but this does not necessarily result in inflammasome formation [9]. Consistent with it, recent studies demonstrated that protein folding stress potentiates NLRP1 and CARD8 inflammasome activation [33,41]. NT degradation is accelerated by several agents that interfere with protein folding, such as aminopeptidase inhibitors. However, these agents alone do not trigger inflammasome formation because the released CT fragments are physically sequestered by DPP9 [41]. In addition, DPP9 aberrant binding to NLRP1 contributes to the pathogenesis of several inflammatory diseases [43,44].

In this study, we demonstrate that SFTSV infection activates the human NLRP1 inflammasome in primary keratinocytes and the CARD8 inflammasome in macrophages in a similar manner by targeting the ternary inhibitory complex, respectively. Infection with SFTSV led to reduction of NLRP1/CARD8 NT and MG132 restored NLRP1 NT, indicating that N-terminal functional degradation occurs in a proteasome-dependent manner during activation of the NLRP1 and CARD8 inflammasome. Unlike protease-mediated cleavage, SFTSV does not have a protease. Thus, SFTSV infection may trigger an endogenous proteasomal degradation pathway that destabilizes NLRP1/CARD8 NT and is cell-type dependent, although the specific mechanism remains to be elucidated. Furthermore, we found that NSs promote NLRP1 and CARD8 inflammasome activation. The NSs interact with both NLRP1 and CARD8 via their FIIND domain, suggesting that DPP8/9 are likely to compete for binding; on the other hand, NSs promote the degradation of DPP8 and DPP9; both mechanisms contribute to the more efficient destabilization of the DPP8/9 ternary complex. In addition, NSs was likely to promote the degradation of DPP8 and DPP9 through the autophagy pathway. Previous studies have demonstrated that NSs could induce autophagy [45,46]. Thus, at least two separate (but perhaps related) danger signals, one that promotes NT degradation and a second that destabilizes the DPP9 ternary complexes, contribute to NLRP1/CARD8 inflammasome activation by SFTSV.

A few studies reported that SFTSV activates the NLRP3 inflammasome [32,34,47]. The mechanism of activation is thought to be initiated by mitochondrial DNA (mtDNA) during SFTSV infection [32,47]. Consistent with this, our study showed that SFTSV activates not only the NLRP3 inflammasome but also the CARD8 inflammasome in macrophages. Moreover, CARD8 deletion potentiates SFTSV replication. This could occur as a result of CARD8-mediated pyroptosis inhibiting viral spread. Thus, CARD8 might play even more major role in SFTSV-induced inflammasome activation compared to NLRP3. Several studies suggested that CARD8-dependent GSDMD pore formation contributes to NLRP3 inflammasome, which also offers an explanation for CARD8-dependent release of IL-1β [15,48]. Future studies may be required to distinguish the relationship between CARD8 and NLRP3.

In conclusion, our study demonstrated that SFTSV triggered the NLRP1 inflammasome in primary keratinocytes and CARD8 inflammasome in macrophages, respectively. Thus, we found a novel mechanism of viral protein activation of NLRP1 and CARD8 by targeting the DPP9-mediated ternary complex. In addition, this study may provide a better understanding of the clinical features of severe SFTS and potential therapeutic strategies to ameliorate the outcomes of SFTS.

## Materials and methods

### Cell lines

HEK293T, Hela, and A549 cells were cultured in DMEM (Gibco, Beijing, China) supplemented with 10% fetal bovine serum (FBS; Gibco, Auckland, New Zealand) and 1% streptomycin-penicillin (5%) at 37°C under a humidified atmosphere containing 5% CO_2_. THP-1 cells were cultured in RPMI-1640 media supplemented with 10% FBS. THP-1 cell differentiation was induced by incubation with 100 ng/ml phorbol 12-myristoyl 13-acetate (PMA) for 24 h, and cells were cultured without PMA for 24 h. Primary keratinocytes were purchased from Regenovo Biotechnology (RB010102) and maintained in KGM Gold BulletKit medium (Lonza, 00192060). Stable cell lines were generated using a standard selection protocol with puromycin (2 μg ml^−1^), hygromycin (400 μg ml^−1^). For generation of knockout cells by the CRISPR–Cas9 system, lentiviruses expressing Cas9 and the desired sgRNA with a GFP selection marker were generated and used to infect primary keratinocytes. GFP positive cells were sorted by flow cytometry (BD FASCAria Fusion) and validated for specific gene knockout by immunoblotting with specific antibodies at 48 h after infection. Sequences of the sgRNA species are 5′-GATAGCCCGAGTGACATCGG-3′ for h*NLRP1*, 5′-TCGCCAATAAAGCGCACTCC-3′ for h*NLRP1*.

### Viruses

SFTSV (strain JS2011-013-1) was utilized in this study and was amplified with a standard protocol by infecting Vero cells as previously described [34]. Viral titer was determined by plaque assay. Briefly, Vero cells were cultured in a 12-well plate at a density of 2 × 10^5^ cells/well and infected with 200 μl 10-fold serially diluted virus solution for 2 h. Then the cells were washed and replenished with plaque medium supplemented with 1% carboxyl methylcellulose. After incubation for 7 days, the infected cells were fixed with 4% formaldehyde and stained with 0.5% crystal violet for plaque assay.

### Antibodies and reagents

Primary commercial antibodies used in this study include: Anti-NLRP1 N-terminal antibody (AF6788) (R&D Systems), Anti-NLRP1 C-terminal antibody (#56719), anti-GSDMD (#36425), anti-pro-IL-1β (#12703), anti-cleaved IL-1β (#83186), anti-V5 (#13202 and #80076), anti-HA (#3724), anti-Flag (#8146), anti-p-p38 (#36425) (Cell Signaling Technology), Anti-ASC (sc-271054), anti-caspase-1 (sc-22613) (Santa Cruz Biotechnology), Anti-DPP9 (ab42080), anti-GSDMD (ab21503), anti-CARD8 C-terminal (ab24186), anti-CARD8 N-terminal (ab194585) (Abcam), Anti-p38 (T55600, Abmart). Primary antibodies specific to SFTSV NP were from our laboratory. Anti-NLRP1 (12256-1-AP), anti-DPP8 (12752-1-AP), anti-NLRP3 (19771-1-AP), anti-MYC (60003-2-Ig) anti-HA (66006-2-Ig), and anti-Flag (80010-1-RR) were purchased from Proteintech. MG132 (AbMole, M1902), VbP (ApexBio, B3941), doxycycline (MCE, HY-N0565), puromycin (MCE, HY-K1057), SB202190 (MCE, HY-10295), Doramapimod (MCE, HY-10320), MLN4924 (MCE, HY-70062), Phosphatase inhibitor cocktail (M7528, AbMole), Lipofectamine 3000 (Thermo Fisher Scientific, L3000015), DSS (Thermo Fisher Scientific, A39267), protease inhibitor cocktail (AbMole, M5293), PMA (Sigma-Aldrich, 16561-29-8), polybrene (Sigma-Aldrich, TR-1003), Pierce IP Lysis Buffer (Thermo Fisher Scientific, 87787), PVDF membrane (Millipore, IPEL00010), and BCA Protein Assay Reagent Kit (Takara, T9300A) were also utilized.

### RNA extraction and quantitative RT-PCR

Total RNA was extracted with Trizol Reagent (Invitrogen, Carlsbad, CA) and the cDNA was synthesized using High Capacity cDNA Reverse Transcription Kit (Invitrogen, Carlsbad, CA). RT-qPCR assays were performed using a ChamQ Blue Universal SYBR qPCR Master Mix (Vazyme, Nanjing, China). Relative mRNA concentrations were calculated by the 2 − ΔΔCt method, normalizing with GAPDH. The sequence of the primers: *SFTSV L* 5′-AGTCTAGGTCATCTGATCCGTTTAG-3′ and 5′- TGTAAGTTCGCCCTTTGTCCAT-3′; *SFTSV M* 5′-AAGAAGTGGCTGTTCATCATTATTG-3′ and 5′- GCCTTAAGGACATTGGTGAGTA-3′; *SFTSV S* 5′-TGTCAGAGTGGTCCAGGATT-3′ and 5′- ACCTGTCTCCTTCAGCTTCT-3′; Human *GAPDH* 5′-GGAGCGAGATCCCTCCAAAAT-3′ and 5′- GGCTGTTGTCATACTTCTCATGG-3′.

### Plasmid constructs

Human NLRP1 (HG30111-NY), ASC (HG11175-CY), pro-Casp-1 (HG11148-NF), GSDMD (HG25207-NF), pro-IL-1β (HG10139-NM), CARD8 (HG12619-NM) and DPP9 (HG11418-NF) plasmid and the ASC-GFP (HG11175-ACGLN) lentiviral vector were purchased from Sino Biological (Beijing, China). The truncates of NLRP1, the lentiviral expression vector, and the CARD8-S297A mutant were constructed by Tsingke Biotechnology. To construct plasmids expressing NP, NSs, Gn, Gc, and Gp corresponding fragments of SFTSV viral cDNA were cloned into pCMV3-HA vector.

### Reconstituted NLRP1/CARD8 inflammasome activation

HEK293T cells were seeded into a 12-well plate (4 × 10^5^ cells/well) and incubated overnight. HEK293T cells were treated with NLRP1 (50 ng) or CARD8 (10 ng), pro-Caspase-1 (10 ng), ASC (10 ng), pro-IL-1β (100 ng), and then transfected with plasmids expressing indicated proteins on the 2nd day. Cells were collected and subjected to immunoblotting for mature IL-1β (p17) at 36 h after transfection.

### Lactate dehydrogenase cytotoxicity assay and ELISA

LDH release was detected with Cytotoxicity assay kit (C20300, Thermo Fisher Scientific). The concentrations of mature human IL-1β in cell culture supernatants were measured with human IL-1 beta Valukine ELISA Kit (VAL101, R&D Systems).

### ASC speck-formation assay

A549-ASC-GFP single clones with low background of ASC aggregation without any stimulation were selected with BD FASCAria Fusion. Cells were seeded into 12-well plates, incubated overnight. Cells infected with SFTSV and then fixed and imaged with fluorescence microscopy at 24 h after infection. For each group, several randomly selected fields with similar cell confluence were analyzed. ASC specks were quantified using ImageJ software. Similar results were obtained in at least three independent experiments.

### ASC-oligomerization assay

The ASC-oligomerization assay was performed as previously described [44]. Briefly, cell pellets were collected and washed in 500 μl cold PBS and then resuspended in PBS. Samples were crosslinked by adding DSS to 4 mM and incubating at 37°C for 30 min with constant mixing. Subsequently, crosslinked pellets were centrifuged at 5000*g* for 5 min and then resuspended in 1 × Laemmli SDS-PAGE buffer for 10 min at 95°C and subjected to immunoblotting analysis.

### Immunofluorescence and confocal microscopy

Cells were fixed with 4% paraformaldehyde for 15 min. After washing with PBS, the cells were permeabilized with 0.2% Triton X-100 for 15 min and blocked with 5% bovine serum albumin for 1 h. The corresponding primary antibodies were incubated overnight at 4˚C and fluorescently labeled secondary antibodies were stained for 1 h. After washing, cells were incubated with 4’, 6-diamidino-2-phenylindole (DAPI; Beyotime, Shanghai, China) for 5 min. The cells were observed using Olympus IX73 fluorescent inverted microscope for immunofluorescence and Leica SP8 confocal laser microscope with 63 × oil objective for confocal microscopy. All image analyses were performed using the software Leica Application Suite X.

### Immunoprecipitation and immunoblotting

For co-immunoprecipitation, 2 × 10^6^ HEK293T cells were transfected with indicated plasmids at a confluence of 90% with Lipofectamine 3000. The cells were washed twice with cold PBS and lysed by Pierce IP Lysis Buffer containing 25 mM Tris-HCl (pH 7.4), 150 mM NaCl, 1% NP-40, 1 mM EDTA, 1% NP-40, 5% glycerol and a protease inhibitor cocktail for 20 min on ice for 48 h after transfection. The cell lysates were then centrifuged at 15,000*g* for 15 min, and the supernatants were subjected to immunoprecipitation with specific antibody overnight at 4°C. Protein A + G agarose were washed three times with lysis buffer and then added to the cell lysis. After 3 h of incubation at 4°C, the beads were washed 3 times with IP lysis buffer and twice with PBS. Subsequently, the beads were resuspended with 2 × loading buffer and boiled for 10 min at 95°C. The immunoprecipitants were used in standard immunoblotting analyses with the indicated specific antibodies.

### Statistical analysis

Most experiments were performed at least three times and statistical analysis was performed using Student’s *t* test or one-way analysis ANOVA with GraphPad Prism Software. *P* values less than 0.05 were considered significant.

## Supporting information

S1 Fig SFTSV activates the NLRP1 inflammasome. (A-B) Production of p17 (A), ASC oligomerization (B) in SFTSV-infected HEK293T cells expressing ASC-Caspase-1- pro-IL-1β. (C) Primary keratinocytes were infected with SFTSV at different MOIs for 24 h, endogenous NLRP1 was detected with Western blot. (D) Expression of HA-NLRP1-Flag in A549 cells treated with lentivirus-mediated *NLRP1* or Vector control. *indicates an unspecific band. (E) A549-HA-NLRP1-Flag cells were infected with SFTSV at different MOIs for 24 h, NLRP1 was detected with Western blot. (F-G) Primary keratinocytes were infected with SFTSV (MOI = 1) and treated with MLN4924 (1 μM) for 20 h, the endogenous NLRP1 (F) was detected with Western blot; IL-1β (G) release in the cell supernatant was measured with ELISA. (H) A549-HA-NLRP1-Flag cells were infected with SFTSV (MOI = 1) in the presence of 5 μM MG132, 10 μM MG132 for 5 h, or 1 μM MLN4924 for 20 h, NLRP1 was detected with Western blot. All data represent three independent experiments and presented as mean±s.d. *P < 0.05, **P < 0.01, ***P < 0.001, ****P < 0.0001, *ns*, not significant. For statistical analysis, two-tailed unpaired Student’s *t*-test in (F).
(TIF)

S2 Fig Supplementary immunoblots for Fig 1. (A) Primary keratinocytes were infected with SFTSV (MOI = 1) for 24 h, or treated with ANS (15 μM) for 5 h, phosphorylated p38 was detected with Western blot. (B-C) Primary keratinocytes were infected with SFTSV (MOI = 1) for 24 h, or treated with ANS (15 μM) for 5 h, in the presence of 20 μM SB or 10 μM Dora, phosphorylated p38 and cleaved GSDMD were detected with Western blot (B); IL-1β (C) release in the cell supernatant was measured with ELISA. All data represent three independent experiments and presented as mean±s.d. *P < 0.05, **P < 0.01, ***P < 0.001, ****P < 0.0001, *ns*, not significant. For statistical analysis, two-tailed unpaired Student’s *t*-test in (C).
(TIF)

S3 Fig SFTSV NSs interacts with NLRP1. (A) Colocation of endogenous NLRP1 and NSs in Hela cells transfected with NSs-HA for 48 h. Scale bar is 20 μm. (B-C) Co-IP assay between NLRP1-Flag and NP-HA (B) or HA-NSs (C) in HEK293T cells transfected with the indicated expression vectors for 48 h. (D) Detection of p17 in ASC-caspase-1-pro-IL-1β HEK293T cells transfected with indicated expression vectors for 36 h.
(TIF)

S4 Fig Supplementary immunoblots for Fig 6. (A) Primary keratinocytes were infected with SFTSV at an MOI of 1 for 24 h in the presence or absence of lentiviruses expressing carrying vector or Flag-DPP9, NLRP1 and cleaved GSDMD were detected with Western blot. (B) Primary keratinocytes were infected with SFTSV at an MOI of 1 in the presence or absence of lentiviruses expressing carrying vector or Flag-DPP9 for indicated time, IL-1β release in the cell supernatant was measured with ELISA. (C) ASC-caspase-1-pro-IL-1β HEK293T cells were infected with SFTSV at an MOI of 0.3 or 1 for 24 h in the presence or absence of Flag-DPP9, NLRP1 and cleaved pro-IL-1β were detected with Western blot. (D) caspase-1-pro-IL-1β HEK293T cells were infected with SFTSV at an MOI of 0.3 or 1 for 24 h in the presence or absence of Flag-DPP9, CARD8 and cleaved pro-IL-1β were detected with Western blot. (E) Detection of cleaved pro-IL-1β in ASC-caspase-1-pro-IL-1β HEK293T cells transfected with indicated expression vectors for 36 h and then treated with CQ (50 μM) for 6 h before harvest. All data represent three independent experiments and presented as mean±s.d. *P < 0.05, **P < 0.01, ***P < 0.001, ****P < 0.0001, *ns*, not significant. For statistical analysis, two-tailed unpaired Student’s *t*-test in (E).
(TIF)
