# Supplementary figures and images for: The non-structural protein of SFTSV activates NLRP1 and CARD8 inflammasome through disrupting the DPP9-mediated ternary complex
Source: PLoS Pathog. 2025 Jul 3;21(7):e1013258. doi: 10.1371/journal.ppat.1013258 (PMC12225885; doi:10.1371/journal.ppat.1013258)

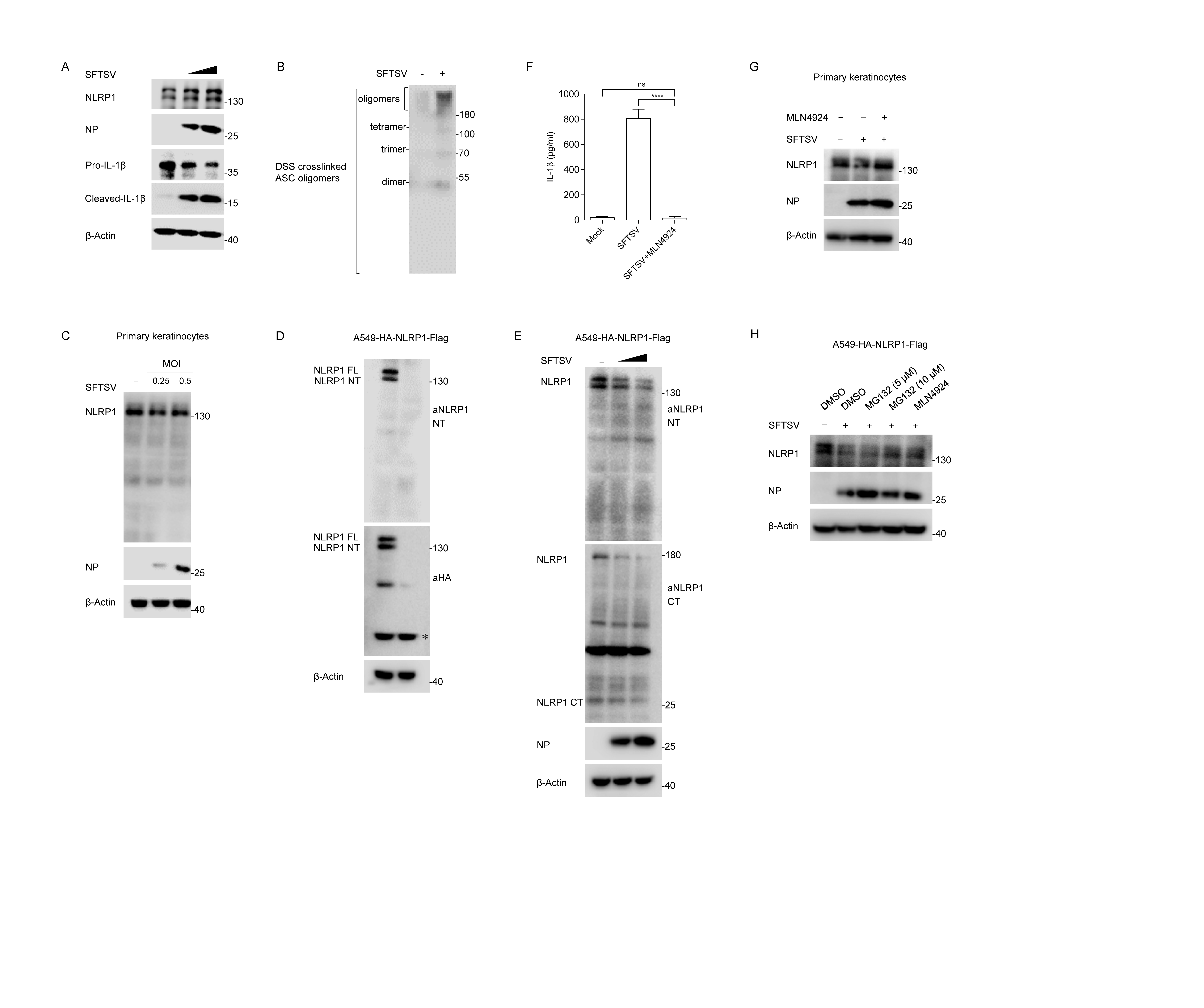

Supplement: S1 Fig — (A-B) Production of p17 (A), ASC oligomerization (B) in SFTSV-infected HEK293T cells expressing ASC-Caspase-1- pro-IL-1β. (C) Primary keratinocytes were infected with SFTSV at different MOIs for 24 h, endogenous NLRP1 was detected with Western blot. (D) Expression of HA-NLRP1-Flag in A549 cells treated with lentivirus-mediated NLRP1 or Vector control. *indicates an unspecific band. (E) A549-HA-NLRP1-Flag cells were infected with SFTSV at different MOIs for 24 h, NLRP1 was detected with Western blot. (F-G) Primary keratinocytes were infected with SFTSV (MOI = 1) and treated with MLN4924 (1 μM) for 20 h, the endogenous NLRP1 (F) was detected with Western blot; IL-1β (G) release in the cell supernatant was measured with ELISA. (H) A549-HA-NLRP1-Flag cells were infected with SFTSV (MOI = 1) in the presence of 5 μM MG132, 10 μM MG132 for 5 h, or 1 μM MLN4924 for 20 h, NLRP1 was detected with Western blot. All data represent three independent experiments and presented as mean±s.d. *P < 0.05, **P < 0.01, ***P < 0.001, ****P < 0.0001, ns, not significant. For statistical analysis, two-tailed unpaired Student’s t-test in (F). (TIF) [file ppat.1013258.s001.tif]

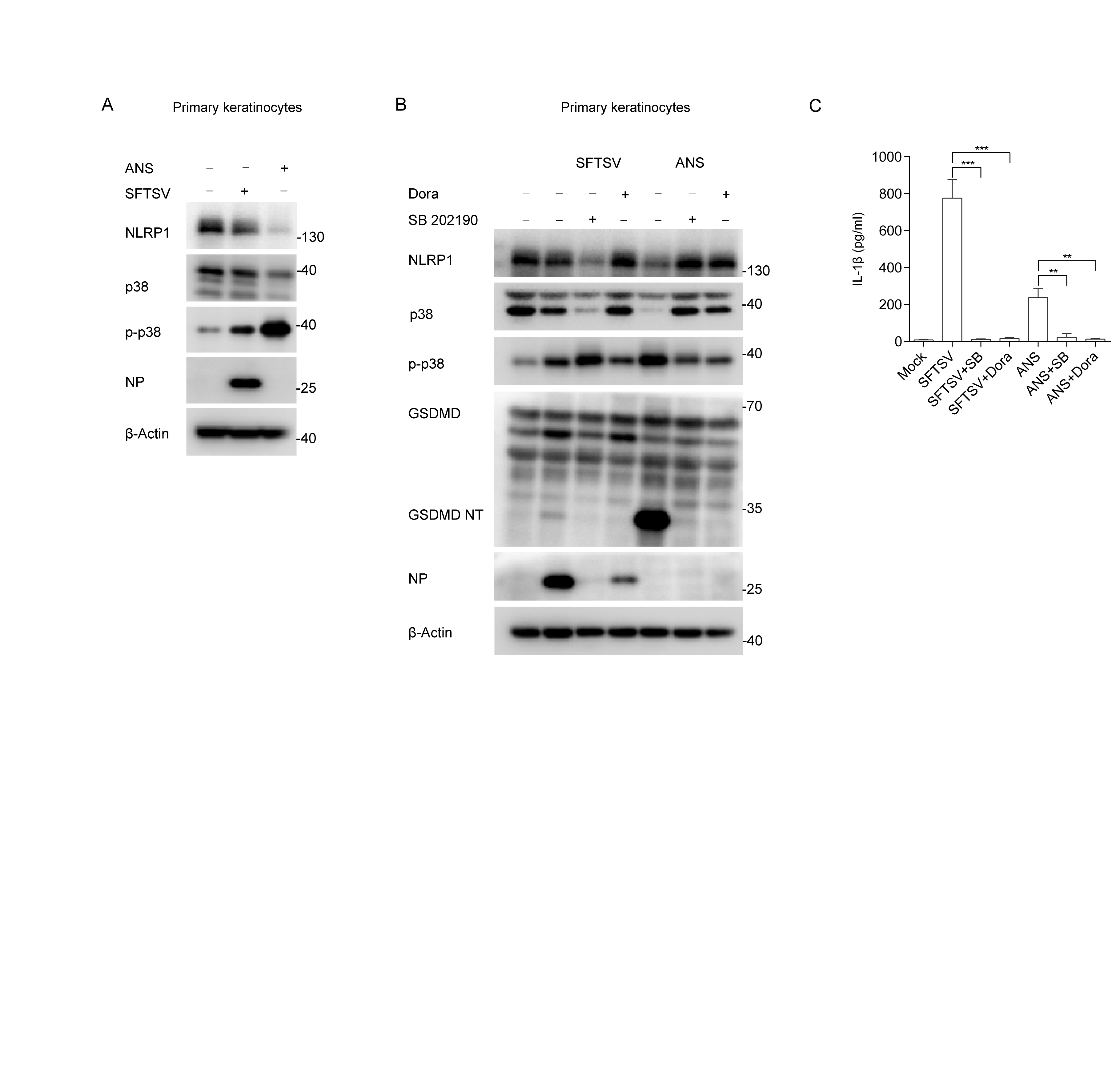

Supplement: S2 Fig — (A) Primary keratinocytes were infected with SFTSV (MOI = 1) for 24 h, or treated with ANS (15 μM) for 5 h, phosphorylated p38 was detected with Western blot. (B-C) Primary keratinocytes were infected with SFTSV (MOI = 1) for 24 h, or treated with ANS (15 μM) for 5 h, in the presence of 20 μM SB or 10 μM Dora, phosphorylated p38 and cleaved GSDMD were detected with Western blot (B); IL-1β (C) release in the cell supernatant was measured with ELISA. All data represent three independent experiments and presented as mean±s.d. *P < 0.05, **P < 0.01, ***P < 0.001, ****P < 0.0001, ns, not significant. For statistical analysis, two-tailed unpaired Student’s t-test in (C). (TIF) [file ppat.1013258.s002.tif]

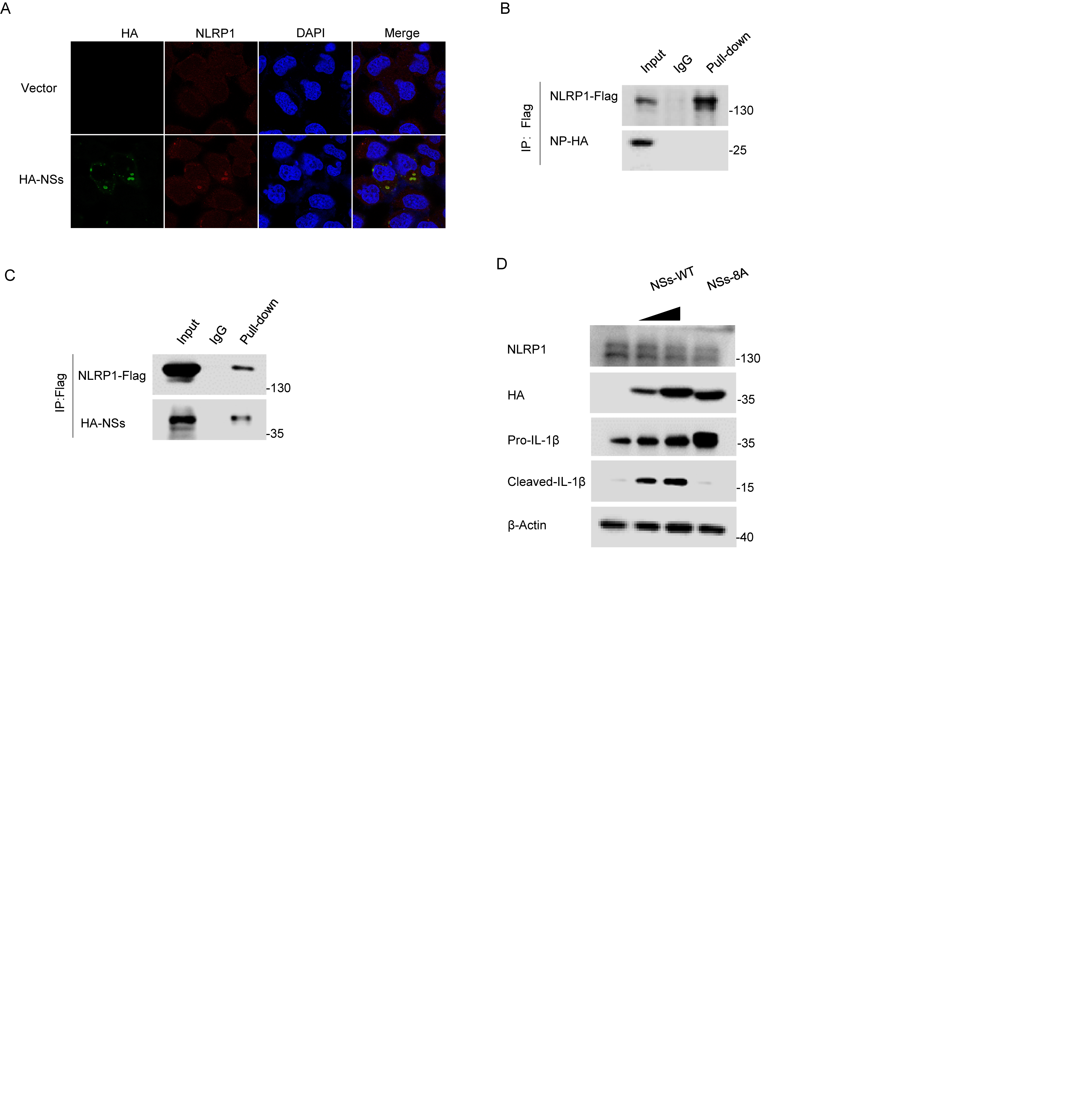

Supplement: S3 Fig — (A) Colocation of endogenous NLRP1 and NSs in Hela cells transfected with NSs-HA for 48 h. Scale bar is 20 μm. (B-C) Co-IP assay between NLRP1-Flag and NP-HA (B) or HA-NSs (C) in HEK293T cells transfected with the indicated expression vectors for 48 h. (D) Detection of p17 in ASC-caspase-1-pro-IL-1β HEK293T cells transfected with indicated expression vectors for 36 h. (TIF) [file ppat.1013258.s003.tif]

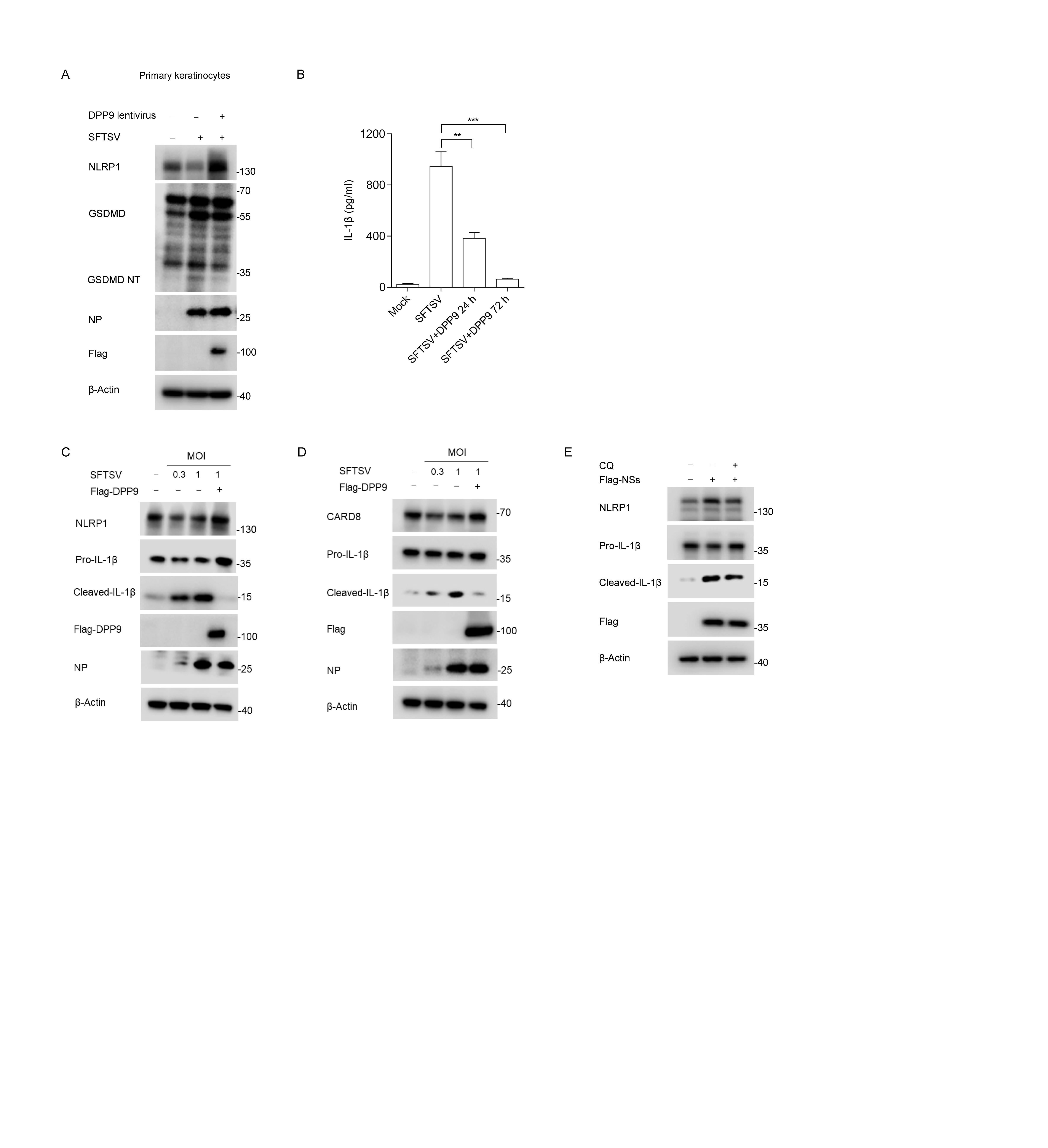

Supplement: S4 Fig — (A) Primary keratinocytes were infected with SFTSV at an MOI of 1 for 24 h in the presence or absence of lentiviruses expressing carrying vector or Flag-DPP9, NLRP1 and cleaved GSDMD were detected with Western blot. (B) Primary keratinocytes were infected with SFTSV at an MOI of 1 in the presence or absence of lentiviruses expressing carrying vector or Flag-DPP9 for indicated time, IL-1β release in the cell supernatant was measured with ELISA. (C) ASC-caspase-1-pro-IL-1β HEK293T cells were infected with SFTSV at an MOI of 0.3 or 1 for 24 h in the presence or absence of Flag-DPP9, NLRP1 and cleaved pro-IL-1β were detected with Western blot. (D) caspase-1-pro-IL-1β HEK293T cells were infected with SFTSV at an MOI of 0.3 or 1 for 24 h in the presence or absence of Flag-DPP9, CARD8 and cleaved pro-IL-1β were detected with Western blot. (E) Detection of cleaved pro-IL-1β in ASC-caspase-1-pro-IL-1β HEK293T cells transfected with indicated expression vectors for 36 h and then treated with CQ (50 μM) for 6 h before harvest. All data represent three independent experiments and presented as mean±s.d. *P < 0.05, **P < 0.01, ***P < 0.001, ****P < 0.0001, ns, not significant. For statistical analysis, two-tailed unpaired Student’s t-test in (E). (TIF) [file ppat.1013258.s004.tif]
